# Feasibility study to improve oral health in older adult patients using visiting nursing services: A pilot study

**DOI:** 10.1371/journal.pone.0313817

**Published:** 2024-12-02

**Authors:** Kyoko Noguchi, Ryota Ochiai, Tomoko Akase, Kazuhiko Nishiyama, Setsuko Watabe

**Affiliations:** 1 Department of Nursing, Faculty of Health Sciences, Shonan University of Medical Sciences, Yokohama, Kanagawa, Japan; 2 Department of Nursing, Graduate School of Medicine, Yokohama City University, Yokohama, Kanagawa, Japan; 3 Kinugasa Healthcare Dental and Orthodontic Clinic, Yokosuka, Kanagawa, Japan; University Hospital Cologne: Uniklinik Koln, GERMANY

## Abstract

Among older adults in Japan, those requiring long-term care who use visiting nursing services have particularly poor oral health. Given the importance of oral health, this study aims to evaluate the feasibility of oral health improvement interventions for such older adult patients using visiting nursing services. This study was a single-arm pilot study. The participants were those who provide oral care to older adults who use visiting nursing services, whether the patients themselves or their family members. Participants implemented oral care appropriate to the patient’s oral environment at least once a day for four weeks. Feasibility assessment consisted of the recruitment, completion, and compliance rates. Changes in the oral environment were measured using the Oral Health Assessment Tool-Japanese (OHAT-J), and changes in scores were assessed over the study period. The study was conducted across three visiting nursing stations, with 52 participants (a recruitment rate of 73.2%). Of these, 42 participants completed the final questionnaire (a completion rate of 80.8%). The compliance rate was 64.3%. The mean OHAT-J score was 4.5 (*SD* 2.3) pre-intervention, 3.7 (*SD* 2.0) at one week post-intervention, and 3.6 (*SD* 2.2) at four weeks post-intervention (*p* < 0.001), indicating a significant positive trend. The feasibility of this intervention was generally satisfactory, and the results showed that the oral environment was improved. Future studies with a larger scale and higher level of evidence should be conducted to evaluate the effectiveness of the intervention.

## Introduction

Maintaining healthy oral function is an important issue, given that oral function contributes to eating, speaking, and aesthetics and affects people’s quality of life [1]. Failure to maintain healthy oral function can lead to dental diseases such as dental caries and periodontal disease, which in turn can reduce quality of life [1]. Additionally, poor oral function can affect systemic health, such as its association with the development of diabetes, dementia, cardiovascular disease, respiratory disease, and cancer [2–7].

One way to prevent these diseases is to implement oral health behaviors that support the maintenance of good oral hygiene. Oral health behaviors comprise three components: *oral hygiene behavior*, *food choice and food acceptance behavior*, and *acceptance behavior for dental care* [8]. In other words, oral health behaviors refer to individual behaviors and habits that maintain oral health and prevent disease. Barriers to maintaining oral hygiene are likely to arise as people age due to a decline in the functions necessary to maintain oral hygiene, such as a decrease in self-cleaning due to a decrease in saliva volume. Declining physical and cognitive functions will also affect the ability to implement oral health behaviors.

Given that Japan’s aging rate in 2022 was 29.0% and is continuing to rise [9], improving older oral health is of considerable importance. In 1989, the Japanese government adopted the “8020 movement,” which aimed to improve the oral health of older adults with the slogan, “Keep at least 20 teeth at age 80.” The achievement rate for improving care in this population reached 50% in 2016, compared to less than 10% at the beginning of the movement [10]. At the same time, the number of older adults with periodontal pockets larger than 4 mm due to caries and periodontal disease has been shown to increase with age [10]. Although this movement policy has been taken, it was reported that 64.3% of older adults in Japan who need care require, needed dental or oral care, but only 2.4% have received dental care [11]. This suggests that older adults with care needs are not receiving dental visits even though they require them. Policies to expand on-site dental care are thus underway [12] but are still in the process of development.

In Japan, the insurance system for visiting nursing services is divided into long-term care insurance [LTCI] and medical insurance, with approximately 70% of users falling under the LTCI system [13]. The LTCI-certified care needs are divided into seven levels: support need levels 1 and 2 and care need levels 1–5 [14]. The higher the level, the more total daily estimated care minutes [14]. Most individuals at care need level 5 are bedridden [15]. Those requiring care need level 3 or higher account for about 40% of Japan’s visiting nursing users, as a large proportion of such individuals need assistance in daily living [13]. Prior research observed that older adults who use visiting nursing services in Japan have particularly poor oral health due to their high care need levels, cognitive decline, and family members’ difficulty in performing oral care for older adults [16]. Improving the oral health of older adults who use visiting nursing services is, thus, an important issue.

Visiting nursing services in Japan differ from those in other countries, partly due to differences in healthcare systems. The provision of visiting nursing services in Europe, for instance, focuses on medical care [17], while the extent of services for patients in Japan is based on physical and cognitive aspects [18], including not only medical care but also assistance with daily living [19]. As such, we expect that oral health care support by visiting nurses could improve the oral health of older adult patients who use visiting nursing services. Currently, there are no oral health care programs, either in Japan or internationally, targeting this population.

Thus, this pilot study aimed to evaluate the feasibility of the program to improve the oral health of older adult patients, considering their high care need levels, cognitive function, and family burden. We posit that this study will not only improve the oral health status and behaviors of older adults using visiting nursing services but also emphasize the importance of care and support by visiting nurses.

## Materials and methods

### Design

This single-arm pilot study was conducted from March through December 2023, focusing on patients or their family members who provided oral care for older adult patients using visiting nursing services. At the time of intervention, the researcher and visiting nurses cooperating in the study suggested individual oral care content to participants, whether patients or their family members. Participants then implemented the recommended oral care content for a four-week period. The oral care content was selected from a consensus among dental professionals, oral surgeons, and visiting nurse specialists suggested in a prior study [20]. This study was conducted under the expanded Consolidated Standards of Reporting Trials [CONSORT] [21, 22].

### Participants

The participants were those who provide oral care for older adults who use visiting nursing services, whether the patients themselves or their family members. Participants were recruited from three visiting nursing stations in Japan, which were selected through convenience sampling.

If the oral care provider was a patient themself, the eligibility criteria were as follows: 1) 65 years of age or older, 2) LTCI-certified care need level 3–5 [16], 3) difficulty in walking independently, 4) requiring support in general daily living and assistance in oral care. The exclusion criteria for patients were as follows: 1) Patients with oral diseases (malignant tumors, serious oral ulcers, etc.) and 2) those who were indicated by visiting nurses cooperating in the study as being unable to perform oral care due to their declining physical function, declining cognitive function, or a rapid decline in their general condition.

If the oral care provider was a family member, the eligibility criteria were as follows: 1) the family member had to be at least 20 years old, 2) the family member should have been implementing care for older adult patients requiring care need level 3 or more, and 3) the family member judged by the cooperator to be able to implement the oral care. Participants who were judged by the cooperators (visiting nurses) to be 1) significantly impaired in physical and cognitive functions and 2) unable to implement oral care were excluded.

### Sample size

Sample sizes ranging from 10 to 40 per group were considered reasonable for testing different possibilities in the context of pilot studies [23]. In addition, a repeated measures one-way analysis of variance (ANOVA) was planned to be used to assess changes in patients’ oral environments before and after the intervention. The sample size was calculated using G*Power, assuming an effect size of 0.25, an alpha error level of 0.05, and a power level of 0.8. A total of 28 participants was considered necessary [24]. Accordingly, the sample size for this study was set to 40 participants. Sampling concluded upon reaching the predetermined target number of cases.

### Intervention

The intervention was conducted at a single time point by the researcher and visiting nurses cooperating in the study. The intervention consisted of the following three procedures (S1 Fig):

1. Evaluation of the participants’ usual oral care by the researcher using the questionnaire
2. Assessment of older adult patients’ oral health by the researcher with direct observation
3. Determination of the oral care contents by researchers, cooperators, and participants, considering the situations described in 1) and 2).

The oral care contents comprised eight domains adapted from the study by Noguchi et al. [20]: *preparation for oral care*, *basic oral care*, *tongue brushing*, *denture care*, *management of oral symptoms*, *care for individuals experiencing a cognitive decline*, *knowledge of prevention of aspiration pneumonia*, and *necessary oral health behaviors*. Participants implemented selected and relevant oral care content during the study period. For instance, if the patients did not have dentures, the section regarding “denture care” was excluded. One consistent aspect across all participants was the inclusion of oral care once a day or more.

### Role of the visiting nurses cooperating in the study

Visiting nurses who agreed to cooperate in this study (hereafter, “cooperator”) participated in the selection of eligible patients or family members for the study and determined the oral care contents for them. Prior to the intervention, a 30-minute lecture for cooperators was conducted at each of the targeted facilities, focusing on the potential oral care content [20]. Supervised by a dentist specializing in dental care for older adults, the lecture was composed of three key domains: methods of intervention, mechanisms of aspiration pneumonia and oral care methods for its prevention, and oral environment observation. Efforts were made to ensure all cooperators and the materials were separately provided to those who could not attend. In addition, cooperators received consultations from participants about the selected oral care during the study period. As a gesture of gratitude for their cooperation, each cooperator received a gift card worth 3,000 yen.

### Data collection

The researcher visited the participants’ residences three times during the study period: at the time of the intervention, one week post-intervention, and four weeks post-intervention, accompanying the cooperator during the provision of visiting nursing services. At each time point, the researcher undertook an oral health assessment and conducted a questionnaire survey regarding difficulties with oral care. All implementation was completed within the contracted hours and carried out in a manner that did not interfere with the provision of conventional nursing care.

After four weeks of intervention, the participants and cooperators were asked to answer a questionnaire to evaluate the intervention. The evaluation included an assessment of whether the intervention was comfortable for the participants as well as the cooperators and the possibility of its continuation.

### Survey items

#### Participant’s background

The participants’ backgrounds were assessed using medical records and the questionnaire at the point of intervention. The following information was collected from their medical records: gender, age, main disease, presence or absence of diabetes [2], presence or absence of dementia [3], LTCI-certified care need level, degree of independence in daily living among the older adults with disabilities [hereafter “daily life independence level”] [25], and the degree of independence in daily living among the older adults with dementia [hereafter “dementia level”] [25].

The LTCI-certified care need levels were determined based on the patient’s physical and cognitive functions [25]. The higher the level, the more daily living assistance required [14, 15, 26]. The daily life independence level represents patients’ physical functioning [25]. The daily life independence levels are designated by the Health, Labour and Welfare Ministry [HLWM] and is divided into 9 levels: independent, J-1, J-2, A-1, A-2, B-1, B-2, C-1, and C-2 [25]. The higher the level, the more daily life support required. The dementia level represents patient’s cognitive functioning [25]. The dementia levels are designated by the HLWM and divided into 10 levels: independent, Ⅰ, Ⅱ, Ⅱa, Ⅱb, Ⅲ, Ⅲa, Ⅲb, Ⅳ, M [25]. The higher the level, the more significant the mental decline [25].

The following information was collected as characteristics of the older adults and oral health behavior from the questionnaire: “Frequency of oral care per day” [27–29], “whether they see a dental visit at least once a year” [27, 28], “Do you go to the dentist as soon as possible if you need to?” [27], “whether they have any teeth remaining” [30], “food intake method” [5], “whether they experience choking while consuming tea or soup [hereafter choking]” [31], and “whether they utilize dentures” [28].

#### Main outcomes: Intervention recruitment, completion, and compliance rates

The main outcomes of the current study were recruitment, completion, and compliance [32–35] rate of the interventions. They were assessed using the questionnaire four weeks after the intervention.

The intervention recruitment rate was defined as the number of participants who met the eligibility criteria and consented to participate in the intervention. The completion rate was defined as the number of participants who participated and their questionnaires until the end of the study (at the end of four weeks). The compliance rate was defined as the number of participants who responded that they were able to perform oral care “at least once a day, every day.” Based on prior studies, the goal of this study was to achieve 70% recruitment, completion, and compliance rates [33–35].

#### Acceptability of the intervention among participants

One of the secondary outcomes of this study was the acceptability of the intervention among participants [32], which was assessed using a questionnaire four weeks after the intervention based on prior research [35]. The questionnaire included the following content: participant satisfaction with the intervention, degree of compliance, perceived ease or difficulty in implementing the oral care, the positive influence of their participation, ease of using the materials, the frequency of problems that occurred during the study period, and the possibility of continuing to proceed with future oral care. Barriers to behavioral compliance (time, mood, physical condition, forgetting, etc.) were extracted from prior studies [36]. The questionnaire asked the following questions utilizing a 4-point scale of 1 (I don’t think so) to 4 (I think so). Finally, we asked the participants to provide a free description of the intervention.

#### Participants’ oral care difficulties

Participants’ oral care difficulties, another secondary outcome of the current study, were assessed at three points: at the intervention, one week post-intervention, and four weeks post-intervention.

Following prior studies [36], this evaluation was based on the Oral Health Assessment Tool [OHAT] items [37] regarding oral symptoms: “oral bleeding,” “mobile teeth,” “dry mouth,” “tongue,” and “halitosis.” Factors that caused difficulties in oral care were “aspiration” [11], “oral care practices” [38, 39], “difficulty in opening the mouth” [40], “physical difficulties” [41], “lack of favoritism for oral care practices” [42], “lack of time” [38, 41–43], “inability to afford for finance” [44], and “refusal to provide oral care” [38, 42]. Of the 13 items, the item on refusal was only asked of family members. Responses to all items were observed on a Likert scale of “1: not at all” to “4: very much.”

#### Acceptability of the intervention among cooperators

We also assessed the acceptability of intervention among cooperators [32]. The assessment was conducted using the questionnaire four weeks after the intervention. The questionnaire content, developed based on prior research [35], was as follows: Ease or difficulty in implementing the intervention, time spent explaining the oral care content to participants, frequency of oral health observations during the study period, the positive influence of participating in the study on the cooperators, frequency of consultations with participants during the study period, and the possibility of continuing the intervention in the future. The questionnaire asked the following questions utilizing a 4-point scale of “1: I don’t think so” to “4: I think so.”

#### Changes in the oral environment

Participants’ oral environment, another secondary outcome of the current study, was assessed at three points: at the intervention, one week post-intervention, and four weeks post-intervention. OHAT was utilized to assess the oral health of the patients [37, 45]. OHAT consists of eight items: “lips,” “tongue,” “gums and tissues,” “saliva,” “natural teeth,” “dentures,” “oral cleanliness,” and “dental pain.” These are rated as “healthy” (0 points), “oral changes” (1 point), and “unhealthy” (2 points). The total score can be calculated by adding up the scores of all eight items, and the possible scores range from 0 to 16 [35]. A Japanese version of the OHAT has been developed (OHAT-J) and was utilized in this study with permission from the developer [46]. The OHAT-J was selected as the outcome of this study because it is one of the most non-invasive measures of oral health and because it is feasible for visiting nurses to reassess it in the future. In this study, one researcher conducted observations of all the patients to minimize inter-rater differences, having learned the direct observation technique from a specialist beforehand.

### Statistical analysis

All participants who completed the four-week oral care implementation were included in the analysis. Descriptive statistics for all survey items were calculated. In the analysis, “daily life independence level” was utilized for analysis converted to binary data “Independence-A” and “B-C.” Also, “dementia level” was converted to “Independence-IIb” and “III-M.” As they represented higher levels, each was considered a factor affecting the oral environment [16].

Differences in participant backgrounds, depending on whether the oral care provider was a family member or the patient themself, were examined using the Mann-Whitney U test, Fisher’s exact test, repeated measures one-way ANOVA, and Friedman test, where applicable. The Friedman test was conducted to examine the changes in the overall OHAT-J score and the scores for the eight items at the three points. The same analysis was conducted for oral care difficulties. SPSS Version 29 was utilized for statistical analysis. All analyses were two-tailed, with a significance level of less than 5%.

The free description field was analyzed using Krippendorff’s [47] content analysis method. After identifying categories based on data similarity, we counted the number of participants with similar descriptions.

### Ethical considerations

This study was conducted with the approval of the Shonan Medical University Ethics Committee (permit number: 22–35). The procedure for obtaining consent was as follows for both cooperators and participants. First, to the cooperators, the researchers explained orally and in writing the purpose, significance, methods, duration, and ethical considerations of the study. They indicated their consent in writing through a signed consent form.

Next, the following procedure was used to obtain consent from the participants. The researcher, along with cooperators, visited the homes of patients who met the eligibility criteria during the time of visiting nursing services. Following the research protocol, patients and family members were provided with information regarding the study, and written consent was obtained from those who agreed to participate. While obtaining consent, we explained that participation was voluntary, no disadvantages would result from non-participation, and responses to the questionnaire would be unsigned. If medical record data is used, we also explained that it should be anonymized using a correspondence table before it is obtained, no non-essential information should be collected, and the utmost efforts should be made to protect personal information. These procedures did not affect the original service provision for visiting nursing users and were completed within the contracted time. Each participant received a gift card worth 3,000 yen as a reward for participating in the intervention.

## Results

### Background of participants and cooperators

A total of 42 participants completed the four-week oral care implementation. Of these, 21 (50.0%) were male, and 21 (50.0%) were female. The mean age of the patients was 85.1 years (*SD* 8.4). The most common underlying disease of the patients was cerebrovascular disease (13 patients, 31.0%). When the oral care provider was a family member, the patients was more inclined to have a high dementia level (III-M) (*p* = 0.015), no remaining teeth (*p* = 0.006), and a low number of remaining teeth (0–9) (*p* = 0.001). In addition, patients tended to experience choking (*p* = 0.029), and the place of dental care tended to be at their own home (*p* = 0.025) (Table 1).

**Table 1:** Participant’s background by oral care provider.

									*n* = 42
			All		Patient		Family		
			(*n* = 42)		(*n* = 21)		(*n* = 21)		
			*n*	*%*	*n*	*%*	*n*	*%*	*p*
Patient gender†		Male	21	50.0	9	42.9	4	19.0	0.181
		Female	21	50.0	12	57.1	17	81.0	
Patient age		(*mean±SD*)	85.1	8.4	81.6	7.8	88.5	7.7	0.003*
Patient’s Disease		Cerebrovascular disease	13	31.0	6	28.6	7	33.3	0.924
		Musculoskeletal disease	10	23.8	4	19.0	6	28.6	
		Malignant neoplasms	7	16.7	4	19.0	3	14.3	
		Respiratory disease	4	9.5	2	9.5	2	9.5	
		Cardiac disease	4	9.5	2	9.5	2	9.5	
		Other	4	9.5	2	9.5	1	4.8	
Co-morbid disease†	Diabetes mellitus	Yes	7	16.7	3	14.3	4	19.0	1.000
		No	35	83.3	18	85.7	17	81.0	
	Dementia	Yes	12	28.6	4	19.0	8	38.1	0.306
		No	30	71.4	17	81.0	13	61.9	
LTCI-certified care need levels		Care need level 3	14	33.3	10	47.6	4	19.0	0.076
		Care need level 4	14	33.3	7	33.3	7	33.3	
		Care need level 5	14	33.3	4	19.0	10	47.6	
daily life independence level†		Independent-A	12	28.6	9	42.9	3	14.3	0.085
		B-C	30	71.4	12	57.1	18	85.7	
dementia level†		Independent-II b	30	71.4	19	90.5	11	52.4	0.015*
		III-M	12	28.6	2	9.5	10	47.6	
Presence of remaining teeth†		Yes	29	69.0	19	90.5	10	47.6	0.006*
		No	13	31.0	2	9.5	11	52.4	
Number of remaining teeth		0–9	22	52.4	5	23.8	17	81.0	0.001*
		<19	8	19.0	6	28.6	2	9.5	
		20 or more	12	28.6	10	47.6	2	9.5	
Food intake method†		Oral intake	37	88.1	19	90.5	18	85.7	1.000
		Other	5	11.9	2	9.5	3	14.3	
Choking†		Yes	22	52.4	7	33.3	15	71.4	0.029*
		No	20	47.6	14	66.7	6	28.6	
Dentures†		Yes	24	57.1	9	42.9	15	71.4	0.118
		No	18	42.9	12	57.1	6	28.6	
Frequency of oral care†		0 times a day	10	23.8	3	14.3	7	33.3	0.277
		Once a day or more	32	76.2	18	85.7	14	66.7	
		Once a day or less	20	47.6	10	47.6	10	47.6	1.000
		Twice a day or more	22	52.4	11	52.4	11	52.4	
Whether they see a dental visit		Yes	12	28.6	6	28.6	6	28.6	1.000
at least once a year†		No	30	71.4	15	71.4	15	71.4	
Form of dental care visits		Going out to see a dentist	11	26.2	9	42.9	2	9.5	0.025*
		Visiting dentist at home	9	21.4	2	9.5	7	33.3	
		No, I don’t see a dentist	22	52.4	10	47.6	12	57.1	
Do you go to the dentist as soon as		Yes	11	26.2	6	28.6	5	23.8	1.000
possible if you need to?†		No	31	73.8	15	71.4	16	76.2	
If the oral care provider is a family member, family background									
Family gender		Male					9	42.9	
		Female					12	57.1	
Family age		(*mean±SD*)					68.6	11.9	
Relationship to patient		Child					13	61.9	
		Spouse					7	33.3	
		Grandchildren					1	4.8	

*SD* = standard deviation

* *p*<0.05

† Fisher’s direct test

There were 27 visiting nurses cooperating in this study, of whom 26 responded to the questionnaire; one could not respond owing to personal reasons. The mean number of years of nursing experience was 25.9 years (*SD* 8.8), and the mean number of years of visiting nursing experience was 10.4 years (*SD* 7.1).

### Main outcomes

Of the 71 participants meeting eligibility criteria, 52 gave their consent (recruitment rate 73.2%). The 19 participants who did not consent included *refusal to participate* (*n* = 11), *hospitalization or institutionalization before participation* (*n* = 5), moving (*n* = 2), and contracting COVID-19 (*n* = 1).

**Fig 1:**
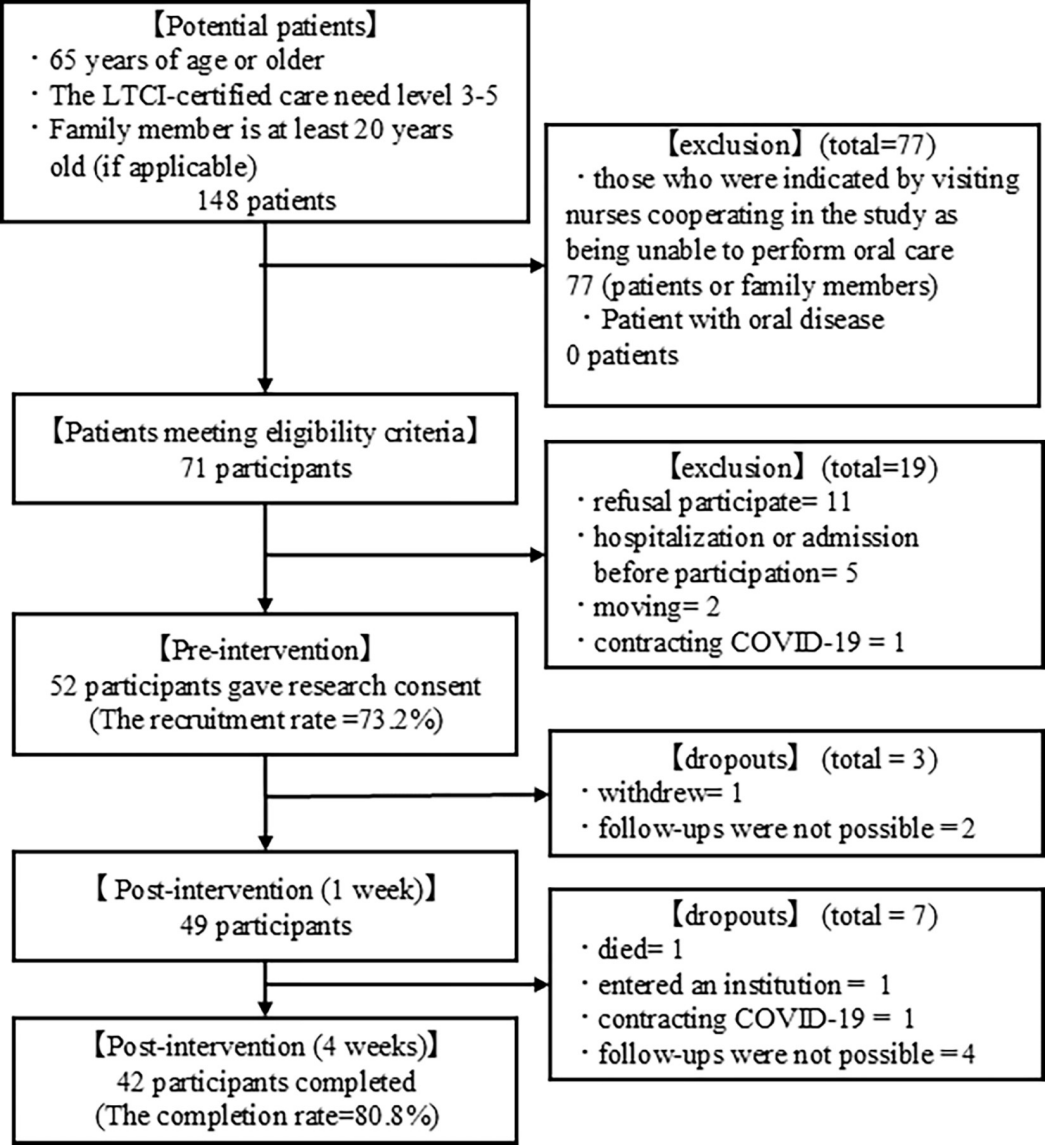
Participant flow. The patients: Older adult using visiting nursing services. The participants: Those who provide oral care for older adults who use visiting nursing services, whether the patients themselves or their family members.

Of the 52 participants, 42 completed the four-week oral care implementation (completion rate was 80.8%) (Fig 1). There were 10 dropouts, of which “one patient died as a result of severe pneumonia caused by COVID-19,” “one entered an institution as a result of difficulty in caring for the patient at home owing to deterioration of cognitive function,” “one could not complete the survey due to COVID-19 contraction,” and “one withdrew.” Six were unable to follow up due to visit time changes.

Of the 42 participants who completed the oral care implementation, 27 reported that they performed the recommended oral care every day (compliance rate of 64.3%). Among them, 16 (76.2%) stated they performed oral care by themselves, and 11 (52.4%) stated that family members performed it for them (Table 2).

**Table 2:** Acceptability of the intervention among participants.

								*n* = 42
		All		Patient		Family		
		(*n* = 42)		(*n* = 21)		(*n* = 21)		
		*n*	*%*	*n*	*%*	*n*	*%*	*p*
Satisfaction with the intervention	Satisfied	27	64.3	14	66.7	13	61.9	0.255
	Somewhat satisfied	13	31.0	5	23.8	8	38.1	
	Somewhat dissatisfied	2	4.8	2	9.5	0	0.0	
	Dissatisfied	0	0.0	0	0.0	0	0.0	
Is this intervention useful?	Useful	25	59.5	12	57.1	13	61.9	0.694
	Somewhat useful	13	31.0	6	28.6	7	33.3	
	Somewhat not useful	3	7.1	2	9.5	1	4.8	
	Not useful	1	2.4	1	4.8	0	0.0	
How much could I do during the period	Every day	27	64.3	16	76.2	11	52.4	0.157
I could do it?	4 or 5 times a week	9	21.4	2	9.5	7	33.3	
	1 or 2 times a week	6	14.3	3	14.3	3	14.3	
	Could not do it	0	0.0	0	0.0	0	0.0	
Ease of doing the oral care actions	Easy	27	64.3	17	81.0	10	47.6	0.054
	Somewhat easy	8	19.0	1	4.8	7	33.3	
	Somewhat difficult	4	9.5	1	4.8	3	14.3	
	Difficult	3	7.1	2	9.5	1	4.8	
Ease of using materials	Easy to use	16	38.1	5	23.8	11	52.4	0.093
	Somewhat easy to use	12	28.6	9	42.9	3	14.3	
	Somewhat difficult to use	8	19.0	3	14.3	5	23.8	
	Difficult to use	6	14.3	4	19.0	2	9.5	
The barriers to behavioral compliance	Lack of time	2	4.8	0	0.0	2	9.5	0.041*
	Mood/energy	4	9.5	4	19.0	0	0.0	
	Physical condition	8	19.0	4	19.0	4	19.0	
	Forgetfulness	5	11.9	1	4.8	4	19.0	
	N/A (no disability)	20	47.6	12	57.1	8	38.1	
	Other	3	7.1	0	0.0	3	14.3	
Frequency of oral problems	Never happened	34	81.0	16	76.2	18	85.7	0.532
	Happened 1 or 2 times	7	16.7	4	19.0	3	14.3	
	Happened once every 2–3 days	0	0.0	0	0.0	0	0.0	
	Happened every time	1	2.4	1	4.8	0	0.0	
Frequency of non-oral problems	Never happened	37	88.1	19	90.5	18	85.7	0.081
	Happened 1 or 2 times	3	7.1	0	0.0	3	14.3	
	Happened once every 2–3 days	2	4.8	2	9.5	0	0.0	
	Happened every time	0	0.0	0	0.0	0	0.0	
Frequency of consulting nurses, dentists, etc.	Did not consult	36	85.7	19	90.5	17	81.0	0.233
	Consulted 1 or 2 times	5	11.9	1	4.8	4	19.0	
	Once every 2–3 days	0	0.0	0	0.0	0	0.0	
	Consulted every time	1	2.4	1	4.8	0	0.0	
The possibility of continuing the intervention	Want to continue	23	54.8	13	61.9	10	47.6	0.781
in the future	Somewhat willing to continue	13	31.0	5	23.8	8	38.1	
	Somewhat unwilling to continue	4	9.5	2	9.5	2	9.5	
	Do not want to continue	2	4.8	1	4.8	1	4.8	

* *p*<0.05

### Acceptability of the intervention among participants

The intervention was evaluated by the 42 participants through a questionnaire administered at the end of the study period (Table 2). Forty participants (95.3%) were satisfied (answering “satisfied” + “somewhat satisfied”), while 38 (90.5%) participants found the intervention useful (answering “useful” + “somewhat useful”). Regarding the accessibility of the materials, 28 (66.7%) answered that they were easy to use or somewhat easy to use. Additionally, 36 (85.8%) participants answered that they wanted to continue the oral care content. Based on the results regarding oral care providers, a significant difference was observed regarding barriers to oral care implementation (*p* = 0.041).

In the free-response section, 12 participants responded when the oral care provider was the patient (S1 Table), comprising six categories, with the highest number of statements being from the “Increased awareness of oral care” category. When the oral care provider was a family member, 10 participants responded (S1 Table), comprising four categories, with the highest number of statements being from the “Increased awareness of oral care” category.

### Participants’ oral care difficulties

For all items, most participants indicated that they felt “no difficulty at all” with oral care, and no significant differences in score trends occurred pre-intervention, at one week post-intervention, and at four weeks post-intervention.

### Acceptability of the intervention among cooperators

Eleven cooperators (42.3%) indicated that the intervention was somewhat easy to implement (Table 3) and 18 (69.2%) answered that the time required to explain the oral care content to participants was less than five minutes. Furthermore, 21 (80.8%) expressed that they wanted to continue or were somewhat willing to continue the intervention in the future.

**Table 3:** Acceptability of the intervention among visiting nurses.

		All	
		(*n* = 26)	
		*n*	*%*
Years of experience† (*mean+SD*)	Nurses	25.9	8.8
	Visiting nurses	10.4	7.1
Ease of implementing this intervention	Easy to implementation	0	0.0
	Somewhat easy to implement	11	42.3
	Somewhat difficult to implement	12	46.2
	Difficult to implement	3	11.5
Time required to explain the oral care contents	Less than 5 minutes	18	69.2
to the participants (n = 25)	More than 5 minutes but less than 10 minutes	7	26.9
	More than 10 minutes but less than 15 minutes	0	0.0
	More than 15 minutes	0	0.0
Frequency of oral environment observation	Not done	6	23.1
during the study period	Once a month	5	19.2
	Once every 2 visits	4	15.4
	Every visit	11	42.3
Duration of oral observation (n = 24)	Less than 5 minutes	23	88.5
	More than 5 minutes but less than 10 minutes	1	3.8
	More than 10 minutes but less than 15 minutes	0	0.0
	More than 15 minutes	0	0.0
Did you encounter any problems during the study period?	Occurred	3	11.5
	Somewhat occurred	4	15.4
	Somewhat did not occur	4	15.4
	Did not occur	12	46.2
Did you ever feel that the intervention was not appropriate for the target group?	I think so	2	7.7
	Somewhat I thought	6	23.1
	Somewhat I didn’t think	3	11.5
	I didn’t think so	13	50.0
Is the intervention useful?	Useful	10	38.5
	Somewhat useful	13	50.0
	Somewhat not useful	0	0.0
	Not useful	3	11.5
Did you receive any consultation from the target group during the study period?	No, I was not consulted.	23	88.5
	Consulted 1 or 2 times	3	11.5
	Once every 2–3 days	0	0.0
	Consulted every time	0	0.0
During the study period, did not consult the researcher when a problem occurred (n = 25)	Did not consult	24	92.3
	Consulted 1 or 2 times	1	3.8
	Once every 2–3 days	0	0.0
	Consulted every time	0	0.0
The possibility of continuing the intervention in the future	Want to continue	8	30.8
	Somewhat willing to continue	13	50.0
	Somewhat unwilling to continue	4	15.4
	Do not want to continue	0	0.0

* *p*<0.05

† Kruskal Wallis test

Seven cooperators responded to the free descriptions, which were generated from four categories (S1 Table). The category that received the highest number of descriptions was “Improvements of the intervention.”

### Changes in the oral environment

**Fig 2:**
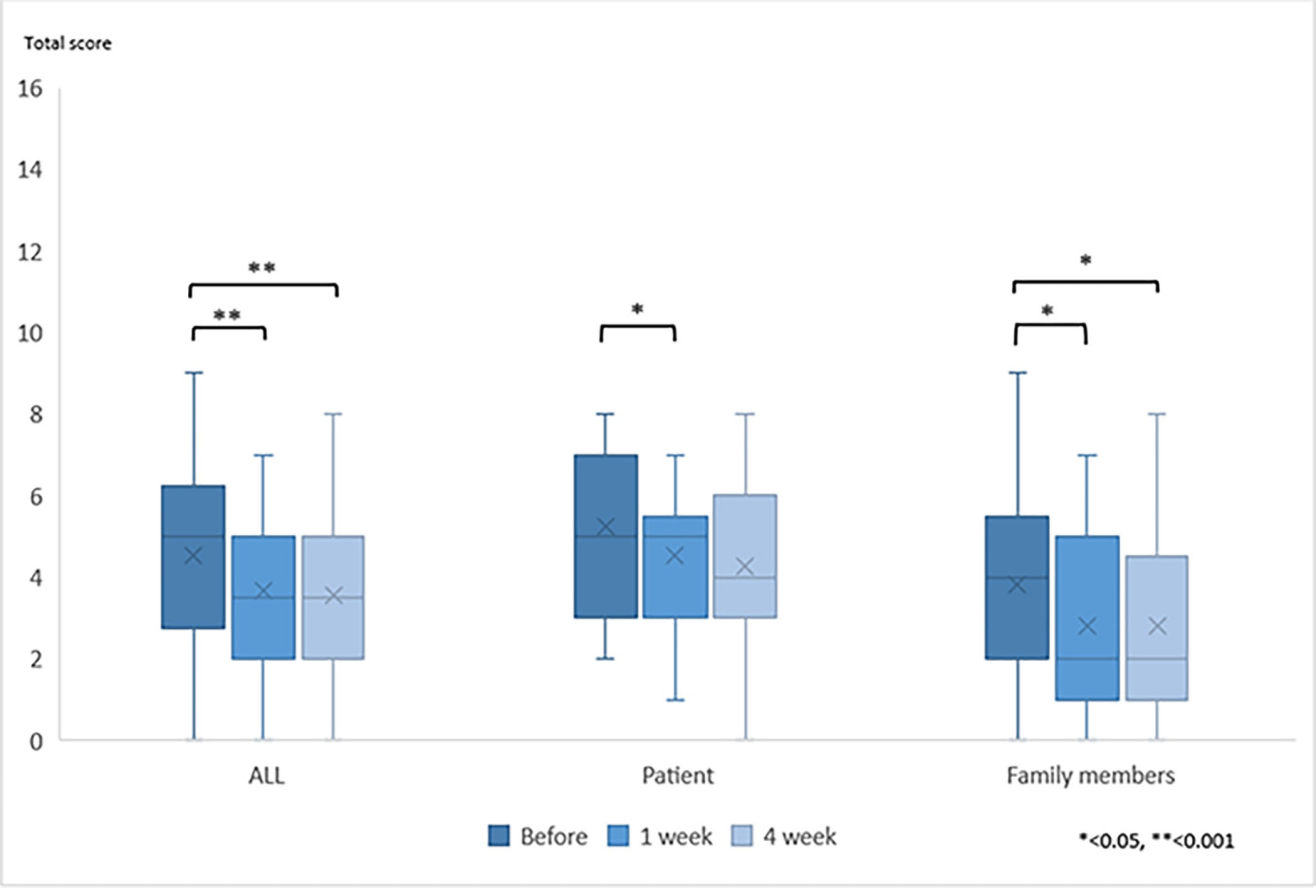
Changes in OHAT-J Score for each group.

The mean OHAT-J score for all patients was 4.5 (*SD* 2.3) pre-intervention, 3.7 (*SD* 2.0) one week post-intervention, and 3.6 (*SD* 2.2) four weeks post-intervention (*p* < 0.001) (Table 4). When the care provider was the patient, the mean OHAT-J score was 5.2 (*SD* 2.0) pre-intervention, 4.5 (*SD* 1.6) one week post-intervention, and 4.3 (*SD* 2.0) four weeks post-intervention (*p* = 0.023). As seen in Table 4, when the care provider was a family member, the scores were 3.8 (*SD* 2.5) pre-intervention, 2.8 (*SD* 2.1) one week post-intervention, and 2.8 (*SD* 2.2) four weeks post-intervention (*p* = 0.001) (Fig 2).

**Table 4:** Changes in the oral environment.

																				*n* = 42
	Pre-intervention						Post-intervention (one week)							Post-intervention (four week)						
	Healthy (0 points)		Oral changes (1 points)		Unhealthy (2 points)		Healthy (0 points)		Oral changes (1 points)		Unhealthy (2 points)			Healthy (0 points)		Oral changes (1 points)		Unhealthy (2 points)		
	*n*	*%*	*n*	*%*	*n*	*%*	*n*	*%*	*n*	*%*	*n*		*%*	*n*	*%*	*n*	*%*	*n*	*%*	*p*
Lips	34	81.0	8	11.4	0	0.0	39	92.9	3	7.1	0		0.0	38	90.5	4	9.5	0	0.0	0.148
Tongue	20	47.6	22	31.4	0	0.0	28	66.7	14	33.3	0		0.0	29	69.0	12	28.6	1	2.4	0.048*
Gums and tissues	15	35.7	17	24.3	10	23.8	18	42.9	15	35.7	9		21.4	15	35.7	17	40.5	10	23.8	0.135
Saliva	13	31.0	29	41.4	0	0.0	21	50.0	21	50.0	0		0.0	25	59.5	17	40.5	0	0.0	0.002*
Natural teeth	21	50.0	15	21.4	6	14.3	21	50.0	15	35.7	6		14.3	20	47.6	15	35.7	7	16.7	0.135
Dentures	27	64.3	4	5.7	11	26.2	28	66.7	3	7.1	11		26.2	29	69.0	3	7.1	10	23.8	0.223
Oral cleanliness	14	33.3	18	25.7	10	23.8	17	40.5	21	50.0	4		9.5	19	45.2	22	52.4	1	2.4	0.001*
Dental pain	39	92.9	3	4.3	0	0.0	41	97.6	1	2.4	0		0.0	40	95.2	2	4.8	0	0.0	0.368
		*mean*	*SD*	*median*	*Q1*	*Q3*		*mean*	*SD*	*median*		*Q1*	*Q3*		*mean*	*SD*	*median*	*Q1*	*Q3*	
OHAT-J score†		4.5	±2.3	5.0	2.8	6.3		3.7	±2.0	3.5	2.0		5.0		3.6	±2.2	3.6	2.0	5.0	<0.001*

OHAT-J = oral health assessment tool J, *SD* = standard deviation; Q1 = 1^st^ quartile; Q3 = 3^rd^ quartile.

*p < 0.*05*.

Freidman Test

## Discussion

This study evaluated the feasibility of a care intervention to improve the oral health of older adult patients who use visiting nursing services. Participants implemented oral care for older adult patients who use visiting nursing services based on a prior study [20] for four weeks. As a result of a four-week continuation of the selected oral care content, high recruitment and completion rates were observed, together with an improvement in oral health. Aspects of feasibility and changes in the oral environment are discussed below.

### Feasibility of this intervention

As a result, the recruitment rate was 73.2%, and the completion rate was 80.8%. These were achieved at the target of 70%. In an intervention targeting care providers of older adult patients with cognitive decline, the completion rate was over 90%, which is higher than in this study [48]. One factor influencing the completion rate in this study could be the presence of difficult-to-follow cases. Data collection was conducted at the time visiting nursing services were provided, considering the burden on participants and cooperators. There were six cases of loss to follow-up in this study, as it may have been difficult to conduct the planned survey owing to the convenience of the patients or their family members. However, the completion rate of this study exceeded 80%, and a certain degree of feasibility can be evaluated.

According to prior studies, compliance rates vary widely, ranging from 23% to 94%, so it cannot be generalized [49]. However, given that interventions targeting older adults in the living community often range from 80% to 90% [50–52], this study’s compliance rate of 64.3% can be said to be lower than in previous studies. The intervention was implemented either by the older adult patients or family members. In the case of family members, the compliance rate was 52.4%. The reasons for this were shown as “forgetting” and “physical condition.” In this study, the researcher visited the participants a total of three times, and the cooperators followed up with the participants during the study period. However, the frequency of follow-up was not limited, which may have influenced the results. A prior study suggests that technology-based interventions can increase compliance rate [53]. For this reason, as in prior studies, periodic phone calls [34], the use of smartphones [53], and other innovations should be made to increase feasibility. Another reason for the low compliance rate among family members was “physical condition”; Japan’s proportion of older adult caregivers is rapidly increasing, with 70% of main caregivers being over 60 years [54] and often having health problems themselves. While focusing on the health status of older adult patients, this intervention did not consider the health status of the family caregivers. Therefore, it is necessary to consider family members’ health status as well.

### Changes in the oral environment

Improvement in the OHAT-J score was observed throughout the older adult patients. This finding is consistent with prior studies on the development of oral care programs for older adults, which have also shown improvements in OHAT scores [48]. The OHAT-J score also improved for oral care providers, both patients themselves and family members, suggesting that a certain clinical benefit can be obtained. However, while the oral environment is one of the causes of aspiration pneumonia [5], as this study aimed to evaluate feasibility, the morbidity of aspiration pneumonia was not included as an outcome. When proceeding to larger-scale implementation and evaluation, morbidity should be set as the main outcome to evaluate the effectiveness of the intervention.

### Limitations

There are several limitations to this study. First, the number of participants is relatively small for multivariate analysis and, therefore, may limit the generalizability of the findings. Future pilot studies and full studies should include larger sample sizes to confirm the results reported herein. In addition, more detailed demographic information on the patients needs to be obtained to better understand the intervention’s applicability to different subgroups. As this study was conducted in one region, regional characteristics were not taken into account. When expanding the scale of the study in the future, we would like to consider regional characteristics.

Moreover, as the study will include multiple facilities, it will be necessary to adjust for bias among facilities by conducting a stratified multilevel analysis for each facility when the main study is recreated using a large-scale survey. We also believe that adjustments will be necessary among care providers. In particular, when oral care providers are family members, many of the older adults requiring and receiving care tend to have impaired physical and cognitive functions, such as low cognitive function and frequent choking. Therefore, bias adjustment is necessary. For example, in a prior study [48], the patients were stratified according to the severity of their cognitive function, and we believe this issue should be considered in this study. Therefore, when moving to a large-scale survey, it is recommended to adjust for bias by stratification and ensure that the number of participants is large enough to withstand analysis.

Secondly, the follow-up period was short. Prior studies have suggested a wide range of follow-up periods from 0.25 months to 2 years [55–57]. In this study, four weeks was set as a reference period, but it was a short period to promote behavior change. Although the feasibility of four weeks and improvement in the oral environment of the subjects were observed in this study, a longer study period is also needed. Finally, applying a randomized controlled trial design would strengthen the evidence provided by the research.

### Future perspectives

Although this is a pilot study to evaluate the feasibility of the program, as a next step, we would like to expand the target population and conduct an RCT. One area for improvement in this study is the need to devise ways to make implementation more convenient for collaborators. One of the considerations is including the delivery time to visiting nurse services. The cooperators were asked to select oral care content and provide consultation information. In this study, the explanations and oral observations took less than five minutes and could be completed without affecting the services for which the participants had contracted. Although it has been suggested that a significant burden on the visiting nurse is avoided and that oral care proves to be useful, only 42.3% of the cooperators indicated that the intervention was easy or somewhat easy to implement in their evaluation of the intervention. This result may reflect a lack of time within the service delivery. The cooperators not only have to implement the care plan within a set time frame, but they also need to respond to unexpected situations. Therefore, those patients who do not normally have an oral care plan and, thus, may have been excluded from the support might have caused implementation difficulties, as nursing care that is not part of the contract may be overlooked due to time limitations.

It is also necessary to consider methods that reduce the burden on visiting nurses. Prior studies have suggested that smartphone pictures can enable remote intraoral assessment [53]. Therefore, it is necessary to devise a way to utilize the smartphone as a communication and consultation tool by recording the oral environment on the smartphone. It was expected that visiting nurses would easily provide reports on the progress of the intervention promotion, and consultations could be made available on time.

## Conclusions

An oral care intervention, derived from previous research, was implemented for four weeks for 42 participants who were conducting the oral care practices for older adults using visiting nursing services in Japan. The feasibility evaluation assessed the recruitment, completion, and compliance rates. For changes in the oral environment, the OHAT-J was used to observe the oral environment at the time of intervention, one week post-intervention, and four weeks post-intervention to confirm the change in scores. The recruitment rate was 73.2%, the completion rate was 80.8%, and the compliance rate was 64.3%. The mean OHAT-J scores were 4.5 (*SD* 2.3) pre-intervention, 3.7 (*SD* 2.0) one week post-intervention, and 3.6 (*SD* 2.2) four weeks post-intervention (*p* < 0.001). The feasibility of this intervention was generally satisfactory, and the results showed that the oral environment was improved. Future studies with a larger scale and higher level of evidence should be conducted to evaluate the effectiveness of the intervention.

## Supporting information

S1 Fig Overall intervention flow. (TIF)

S1 Table Acceptability ratings of interventions. (XLSX)

S1 Data set (XLSX)

S2 Data set (XLSX)
