# Supplementary figures and images for: Feasibility study to improve oral health in older adult patients using visiting nursing services: A pilot study
Source: PLoS One. 2024 Dec 2;19(12):e0313817. doi: 10.1371/journal.pone.0313817 (PMC11611131; doi:10.1371/journal.pone.0313817)

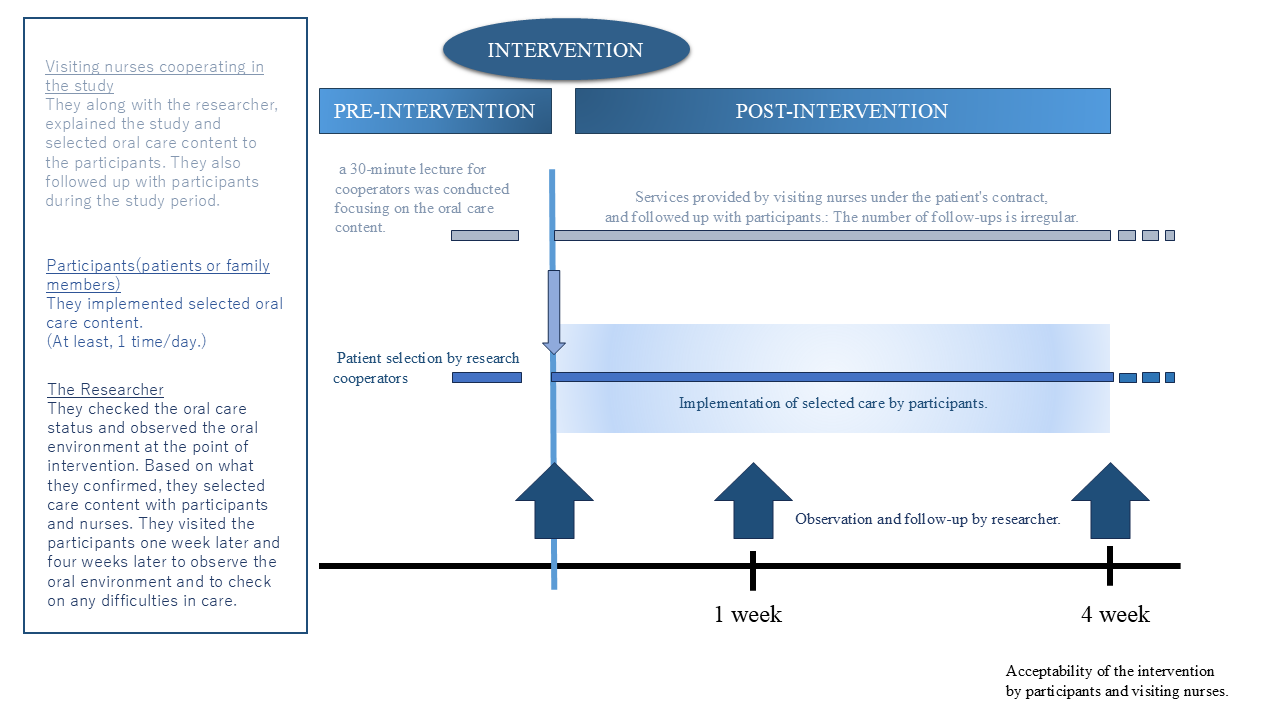

Supplement: S1 Fig — (TIF) [file pone.0313817.s001.tif]
